# Incidence proportion of complex regional pain syndrome (CRPS) after distal radius fracture: a population-based register study

**DOI:** 10.2340/17453674.2026.45443

**Published:** 2026-02-20

**Authors:** Pernille MELBYE, That Minh PHAM, Niels-Peter Brøchner NYGAARD, Carsten Hanshelge KOCK-JENSEN, Per Hviid GUNDTOFT, Bjarke VIBERG

**Affiliations:** 1 Department of Orthopaedic Surgery and Traumatology, Hospital Lillebaelt Kolding – University Hospital of Southern Denmark, Kolding; 2 Department of Orthopaedic Surgery and Traumatology, Aarhus University Hospital, Aarhus; 3 Department of Orthopaedic Surgery and Traumatology, Odense University Hospital, Odense; 4 CRPS center Region of South Denmark, Hospital of Southwest Jutland, Denmark

## Abstract

**Background and purpose:**

One of the most severe complications of distal radius fractures (DRF) is the development of complex regional pain syndrome (CRPS). The incidence proportion (IP) of CRPS following DRF varies widely in the literature. Our aim is to report the incidence proportion of CRPS in DRF patients, subgrouping on age, sex, and treatment choice, and secondarily to assess development over time.

**Methods:**

Data was extracted from the Danish National Patient Register on patients > 18 years diagnosed with a DRF (S525) in the period 1998–2017.

**Results:**

There were 247,128 DRF in 203,533 patients with a mean age of 61 years. 75% were females. Within 1 year, 493 DRF patients developed CRPS corresponding to a 1-year IP of 0.20% and with an incidence density of 0.57/100,000/year. Median time from DRF to diagnosis was 89 days (SD 73). The IP ranged from 0.01% to 0.39% between age groups with the 30–65-year-olds having the highest incidence proportion. The surgically treated group had an IP of 0.31% and the non-surgical group had an IP of 0.17%. CRPS was slightly more common in women than men (0.21% vs 0.16%). We found a decrease in IP after 2010 from 0.24% to 0.14%.

**Conclusion:**

There was a low IP of CRPS diagnosis after DRF treatment with an observed higher IP in the 30–65-year-olds and in surgically treated patients. We consider this to be a minimum IP due to possible undiagnosed cases, but the overall results may be closer to the clinical reality than previous studies.

Distal radius fractures (DRF) are one of the most common fractures [1-4]. The absolute numbers are steadily increasing, driven by the growing elderly population [1,4]. Among the potential complications following a DRF, complex regional pain syndrome (CRPS) stands out as one of the most severe [5,6].

The diagnosis can be based on the Budapest Criteria, introduced in 2004 and validated in 2010, which requires ongoing pain disproportionate to the inciting event, symptoms in at least 3 of 4 categories (sensory, vasomotor, sudomotor/edema, and motor/trophic), and at least 1 objective sign in 2 of these categories. Furthermore, other potential diagnoses must be excluded [7-9].

The incidence density of CRPS varies between 2 and 26/100,000/year and fractures of the extremities account for 40–50% of these cases [10]. The reported incidence density of CRPS following DRF varies significantly in the literature, primarily due to small studies and different criteria for diagnosis, ranging from less than 1% to 32% [6,11]. A recent meta-analysis [12] estimated the incidence density to be 14% after radius fractures. However, this finding is based on studies that utilized varying diagnostic criteria for CRPS, along with issues like selection bias, small sample sizes, and limited follow-up durations. The lack of large, long-term population-based studies and the absence of consensus on diagnostic criteria likely contribute to the discrepancies in reported incidence proportions.

To understand and treat CRPS after DRF, it is essential to determine the extent of the condition. The primary aim of our study is therefore to report the incidence proportion of CRPS in DRF patients with subgrouping on age, sex, and choice of treatment. Secondarily we aim to assess any development over time, especially after the introduction of the Budapest Criteria in Denmark.

## Methods

### Study design

This is a population-based register study of patients with DRF registered in the Danish National Patient Register (DNPR) from 1998 to 2017. Reporting is performed according to the RECORD extension of the STROBE guidelines [13].

### Setting

All Danish citizens are assigned a personal identification number at birth or immigration by the Danish Civil Registration System, known as the civil registry number. This system encodes sex and date of birth. The civil registry number facilitates simple, accurate, and cost-effective individual-level record linkage across all Danish medical databases, ensuring that every patient can be traced until death or emigration [14]. The Danish National Health Service extends healthcare services to all Danish citizens, guaranteeing free access to emergency care, general hospital care, and outpatient visits [15]. The civil registry number enables the retrieval of the complete hospital discharge history for every Danish citizen. Patients with distal radius fractures are, unless they are undiagnosed, managed within the hospital system in Denmark and recorded in the DNPR, as only public hospitals treat patients with acute fractures in Denmark. Patients treated at a Danish Hospital with a DRF are registered and can be traced in the DNPR, as can patients with surgically treated DRF. The registry data include the civil registry number, dates of admission and discharge, and the surgical procedures performed. All discharge diagnoses are assigned by the doctor who discharges the patient [16].

### Participants

All Danish patients aged 18 years of age and above diagnosed with an International Statistical Classification of Diseases version 10 (ICD-10) code for DRF (S525) were extracted from the DNPR. Procedure codes were included to differentiate between surgical and non-surgical treatment. Non-surgical treatment was defined as not having surgery within the first 3 weeks after the diagnosis. The surgical procedure codes were defined as plate (KNCJ65), external fixation (KNCJ25), K-wire (KNCJ45), and “other” (KNCJ35, 55, 75, 85, and 95). The codes we used to determine whether the patients were diagnosed with CRPS were defined as CRPS code (M890), causalgia (G564), posttraumatic dystrophia (T796B) and block of the sympathetic trunk (KTAD00).

### Variables

The age of patients was ascertained based on their social security number and recorded date for the DRF diagnosis code. Incidence density data were stratified by age using 5-year age intervals: from 18–24, to 75–79, and 80+. Sex was classified as male or female based on their social security number. Follow-up period was 1 year during the entire period; we also included 2 and 5 years’ follow up.

### Data source

In 1977, the Danish National Patient Registry (DNPR) was established. It encompasses 99.7% of all hospital discharges in Denmark [14,16]. Reporting is obligatory for all hospitals, both public and private. Recorded data includes diagnosis codes based on the ICD-10 since 1994, and surgical procedure codes according to the Nordic Medico-Statistical Committee (NOMESCO) classification system [17]. The positive predictive value (PPV) for DRFs has not been estimated, but the PPV for a correct primary diagnosis in orthopedic surgery is reported to be 83% [14].

### Bias

We chose to use only the ICD-10 code for DRF (DS525) and not the code for both DRF and ulnar fracture (DS526). Therefore, there is a risk of miscoding as a DRF with a styloid ulna fracture might be recorded other than with DS526. Throughout the study period, we assume that the risk of miscoding has remained constant.

Surgical treatment was specifically defined as any surgery conducted within 21 days of the initial diagnosis. Consequently, the group categorized as surgically treated included cases of DRFs that were initially managed nonoperatively with a cast but within 21 days necessitated surgical intervention due to treatment failure. Any alterations in treatment within the first 21 days could not be evaluated in this study.

Within the DNPR, the differentiation between open and closed fractures is not possible, as Danish healthcare does not use the specific ICD-10 codes for open fractures. As open distal radius fractures are rare, we estimated that the potential impact of bias from open fractures is minimal [18].

### Statistics

Descriptive statistics were used, and results were presented as total counts and percentages. Further statistical analyses were not performed. STATA 18 (StataCorp LLC, College Station, TX, USA) was used to perform the data workup, and Excel (Microsoft Corp, Redmond, WA, USA) was used for graphical displays. The incidence proportion was estimated as the proportion of patients in the study population that developed CRPS, and the incidence density was estimated as the rate of new occurrences of CRPS compared with person-time at risk, with patients being followed from the date of injury until death, emigration, or 1-year after their fracture, whichever came first.

### Ethics, data sharing, use of AI tools, funding and disclosures

Data were stored and accessed through the Danish Health Data Authority’s secure research platform (Forskermaskinen), to which the authors had full access throughout the study. Data access was approved by the Region of Southern Denmark (journal number 20/187). In accordance with Danish legislation, no further ethical approval was required. Data is available upon reasonable request. AI tools were used for spelling and grammar check. The authors declare no conflicts of interest. Complete disclosure of interest forms according to ICMJE are available on the article page, doi: 10.2340/17453674.2026.45443

## Results

**Figure 1:**
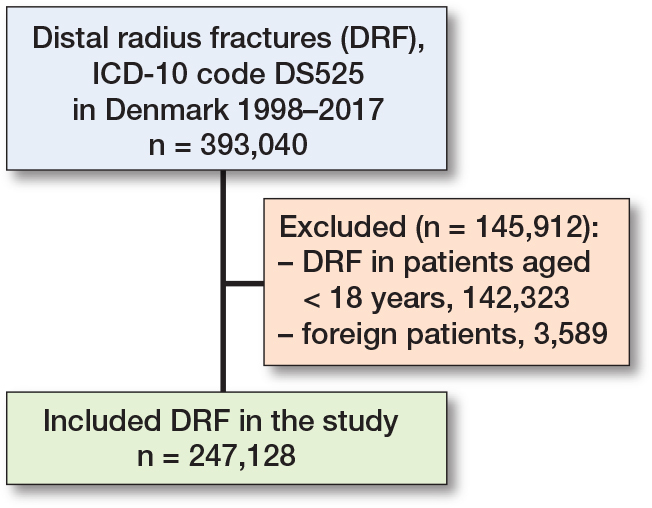
Patient flowchart.

In the 20-year period, there were 247,128 DRFs in 203,533 patients (Figure 1), with a mean age of 61.7 years (SD 19), and 75% were female. Within the 1st year after the DRF diagnosis, 493 patients were diagnosed with CRPS, and there was a minor increase after 2 and 5 years’ follow-up (Table). The median time to CRPS diagnosis was 89 days (SD 73) after the DRF diagnosis within the 1-year follow-up.

**Table:** Characteristics of patients with a distal radius fracture including 1-to-5-year CRPS follow-up timepoints. Values are count (%)

Factor	1-year total	1-year CRPS	2-year total	2-year CRPS	5-year total	5-year CRPS
Total	247,128 (100)	493 (0.20)	232,598	509 (0.22)	190,414	478 (0.25)
Sex						
Male	61,017 (25)	97 (0.16)	57,586	100 (0.17)	47,343	101 (0.21)
Female	186,111 (75)	396 (0.21)	175,012	409 (0.23)	143,071	377 (0.26)
Age						
18–24	12,345 (5.0)	1 (0.01)	11,585	1 (0.01)	9,397	3 (0.03)
25–29	6,787 (2.7)	5 (0.07)	6,428	6 (0.09)	5,306	6 (0.11)
30–34	3,841 (1.6)	10 (0.26)	6,516	11 (0.29)	5,584	17 (0.30)
35–39	8,250 (3.3)	24 (0.29)	7,898	25 (0.30)	6,731	25 (0.37)
40–44	9,868 (4.0)	22 (0.22)	9,395	24 (0.24)	7,952	26 (0.33)
45–49	12,079 (4.9)	37 (0.31)	11,450	40 (0.35)	9,477	38 (0.40)
50–54	17,957 (7.3)	70 (0.39)	16,896	73 (0.43)	13,957	74 (0.53)
55–59	24,906 (10)	92 (0.37)	23,502	98 (0.42)	19,500	91 (0.47)
60–64	27,858 (11)	95 (0.34)	26,267	96 (0.37)	21,467	81 (0.38)
65–69	27,684 (11)	57 (0.21)	25,895	57 (0.22)	20,417	47 (0.23)
70–74	25,587 (10)	42 (0.16)	23,643	41 (0.17)	18,839	32 (0.17)
75–79	23,611 (9.6)	23 (0.10)	22,136	21 (0.09)	18,128	20 (0.11)
80–84	20,227 (8.2)	10 (0.05)	19,113	11 (0.06)	15,917	13 (0.08)
85–89	14,504 (5.9)	3 (0.02)	13,772	3 (0.02)	11,295	3 (0.03)
90–94	6,780 (2.7)	1 (0.01)	6,383	1 (0.02)	5,098	1 (0.02)
95–99	1,688 (0.7)	1 (0.06)	1,571	1 (0.06)	1,241	1 (0.08)
>100	156 (0.1)	0 (0.00)	149	0 (0.00)	108	0 (0.00)
Treatment						
Non-surgical	200,285 (81)	350 (0.17)	189,050	362 (0.19)	156,532	346 (0.22)
Surgical	46,843 (19)	143 (0.31)	43,548	147 (0.34)	33,882	132 (0.39)

**Figure 2:**
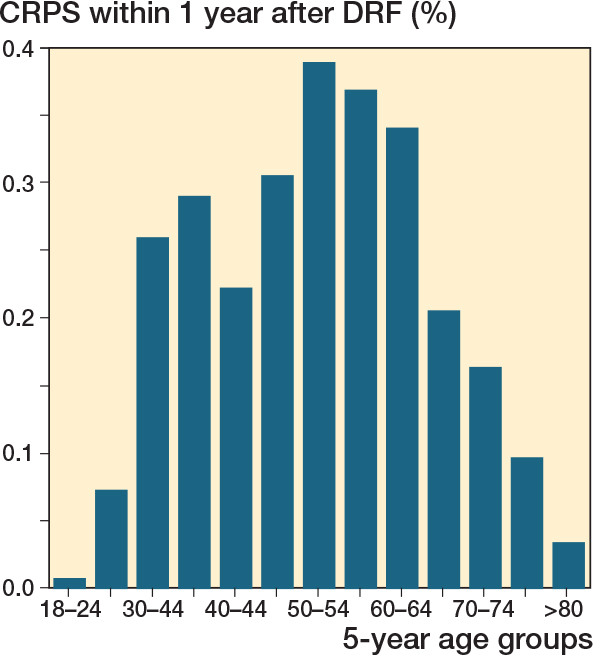
Incidence of CRPS 1 year after DRF divided by age groups.

The overall incidence proportion of CRPS was 0.2% within 1-year follow-up, corresponding to an incidence density of 0.57/100,000/year in the Danish population (see Table). The incidence proportion varied between age groups, ranging from 0.01% to 0.39%, and the highest incidence proportion was observed in the 30–65-year-old group (Figure 2). The incidence proportion increased slightly with longer follow-up: 0.23% with 2-year follow up and 0.25% with 5-year follow up. The incidence proportion was slightly lower in men (0.16%) than in women (0.21%). We observed a higher CRPS incidence proportion among surgically treated DRF (0.31%) than those who were treated non-surgically (0.17%).

**Figure 3:**
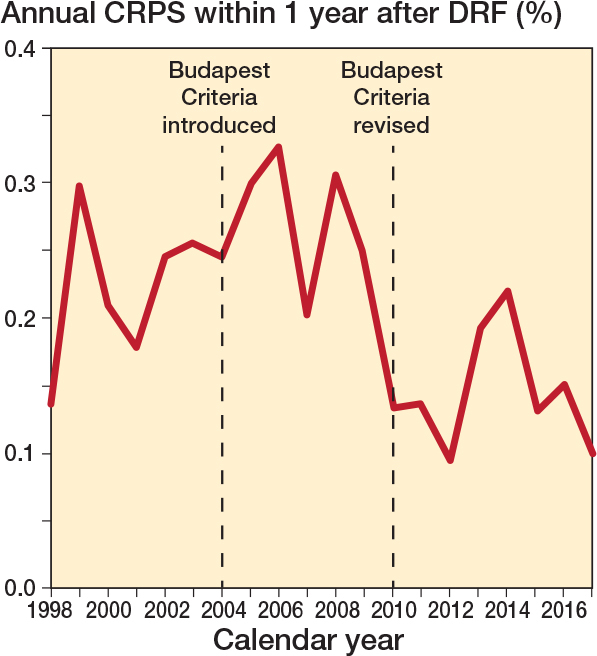
Incidence proportion in percentage of patients diagnosed with CRPS within 1 year after DRF over time. The 1st dashed line (2004) marks when the Budapest Criteria were introduced and the 2nd line (2010) indicates a significant decrease in diagnosed cases (P < 0.05).

When estimating variation over time, the incidence proportion remained almost constant in the years after 2004, when the Budapest Criteria were introduced. However, around 2010, when the criteria were revised, we observed a decrease in CRPS diagnosis. Here we found that the incidence proportion fell from 0.24% before 2010 to 0.14% after 2010 (Figure 3).

## Discussion

We aimed to report the incidence proportion of CRPS in DRF patients, subgrouping on age, sex, and treatment choice, and secondarily to assess development over time. We found an overall CRPS incidence proportion of 0.2% within the 1st year after a distal radius fracture. The highest incidence proportions were observed in individuals aged 30 to 65 years. We also observed a higher incidence proportion of CRPS among surgically treated DRF patients compared with those treated non-surgically.

The incidence proportion observed in our study is considerably lower than that reported in a recent meta-analysis that found a CRPS incidence proportion of 14% following any radius fractures [12]. The meta-analysis included studies written in English on adult patients with fractures of the radius, i.e., not DRF exclusively. The authors caution that their findings should be interpreted carefully, due to selection bias. Some of the studies were based on patients treated in rehabilitation departments. Earlier prospective cohort studies do in general reveal a much higher incidence of CRPS after DRF compared with our study, with incidence proportions ranging from 3.8% to 32% of CRPS after DRF [5,19–21]. They all use different diagnostic criteria for diagnosing CRPS, but only one uses the Budapest Criteria [20], which might be one explanation for the high variability in incidence density among these studies. Further, all the study cohorts are small and the follow-up periods relatively short at 4 to 9 months. One study included only surgically treated patients [20], the others only non-surgically treated patients [5,19,21]. Adding to this selection bias, Jellad et al. [5], who found the highest incidence proportion, included only patients who were sent to a rehabilitation center after experiencing pain [11].

Much lower incidence, similar to ours, is found in 2 population-based studies based on insurance registers: 0.19% based on 59,765 Dutch patients with a DRF in a 2-year period [22] and 0.64% in 172,194 surgically treated DRF patients with 1-year follow-up in the USA [23]. Our study contributes with an even larger study cohort during a 20 year-period including both surgically and non-surgically treated patients. The population-based studies indicate that a coded CRPS diagnosis is rarely seen in patients following a DRF. As the population-based studies, including ours, are performed on a large, unselected group we might assume that this low incidence is closer to the truth incidence of CRPS following DRF. Thus we believe that the large variation in reported CRPS incidence is primarily due to differences in diagnostic criteria, study design, and patient selection, with smaller, highly selected cohorts and less stringent diagnostic tools, tending to overestimate the true incidence compared with large, population-based studies.

The higher incidence of CRPS in the surgically treated patients compared with those treated non-surgically supports earlier findings that more invasive treatments may elevate the risk of CRPS [6,20,23]. Surgical intervention may cause greater tissue damage, increased inflammation, or longer recovery times [12]. CRPS is reported to be more common after open reduction and internal fixation and external fixation, compared with closed reduction and pinning [23]. However, the recent meta-analysis did not find any difference in CRPS in relation to type of treatment [12]. Other factors, such as the severity of the fracture or patient characteristics, may also contribute to the higher CRPS rates after surgery.

One can assume that changes in diagnostic criteria would affect the occurrence of CRPS, but no change was seen after the introduction of the Budapest Criteria in 2004. The criteria were revised and validated in 2010, and we found a significant decrease in incidence density after this year. We hypothesize that it takes years to implement new criteria in clinical practice.

### Limitations

The primary limitation of our study is a possible underreporting of the CRPS diagnosis, which might lead to underestimation of the true incidence density. The diagnosis may be diagnosed but not registered, or patients’ symptoms may be misinterpreted. Because CRPS can be difficult to diagnose, these results might not reflect the actual burden of the condition.

Another limitation is that although a number of patients had bilateral fractures, we analyzed all fractures as individual events even though some correlation exists between bilateral fractures. There is a small risk that this correlation might affect our estimates, but, given the rarity of CRPS, we do not believe that it would profoundly change our results or conclusion. We included only the ICD-10 code for an isolated DRF and not when combined with an ulnar fracture. This could lead to potential bias in both number of DRF and incidence density of CRPS, which might be higher. The ICD-10 codes have not been validated in the Danish National Patient Registry, but we do not expect this to have any impact on the results of this study because the ICD-10 code for CRPS is considered highly specific and is typically used only when the diagnosis is made with a high degree of clinical certainty. We did not include combined radius and ulna fractures which may explain a somewhat lower incidence, as DRF involving fracture of ulna is more severe [24] and might constitute a risk factor for developing CRPS.

The categorization of treatment into surgical and non-surgical groups did not account for changes in treatment approach after 21 days, such as late surgery after initial non-surgical treatment. This simplification may have affected the accuracy of the comparison between treatment groups and their CRPS incidence proportions.

### Strengths

This study is non-selected, population-based, and uses data from the DNPR, which encompasses a larger population over a 20-year period with a large, unselected cohort. This provides a valuable insight into long-term trends in CRPS after a DRF and gives a picture of whether the changes in both clinical practice and changes in diagnostic criteria for CRPS have affected the incidence.

### Conclusion

We found a very low incidence of CRPS after distal radius fracture. CRPS was slightly more common after surgical treatment and in patients aged between 30 and 65 years.

The incidence proportion decreased after 2010, which could be associated with the revision and validation of the Budapest Criteria, a diagnostic tool used in Denmark.

## Notes

PM, BV and TMP proposed the idea of the study. All authors contributed to the development of the protocol. The dataset was provided by BV and PG. BV and PM conducted data analyses. PM wrote the manuscript. All authors contributed to refine the manuscript.

Handling co-editors: Cecilia Rogmark and Jonas Ranstam

*Acta* thanks the anonymous reviewers for help with peer review of this manuscript.
